# The Mouthparts of Female Blood-Feeding Frog-Biting Midges (Corethrellidae, Diptera)

**DOI:** 10.3390/insects14050461

**Published:** 2023-05-13

**Authors:** Stephan Barton, Jonas Virgo, Harald W. Krenn

**Affiliations:** 1 Unit Integrative Zoology, Department of Evolutionary Biology, University of Vienna, Djerassiplatz 1, 1030 Vienna, Austria; stephanbarton@hotmail.com; 2 Department of Animal Ecology, Evolution and Biodiversity, Ruhr-University Bochum, Universitaetsstrasse 150, 44805 Bochum, Germany; jonas.virgo@ruhr-uni-bochum.de

**Keywords:** piercing proboscis, mouthparts, hematophagous, sensilla, Diptera

## Abstract

**Simple Summary:**

Female frog-biting midges exclusively feed on blood from frogs. They are attracted by the calling of male frogs and search for specific feeding sites on their host’s body. To feed, these blood-feeding midges use a very short proboscis that is composed of six piercing structures and an enclosing component. We analyzed the morphology of the mouthparts using SEM and compared these with the well-studied proboscises of other blood-feeding flies. Females of *Corethrella* share more similarities to the very small blood-feeding short-proboscid biting midges, black flies and sand flies than to their more closely related long-proboscid mosquitoes. We interpret our findings in the functional context of a very short piercing proboscis and its possible specialization to pierce frogs.

**Abstract:**

Females of frog-biting midges (Corethrellidae) obtain their blood meals from male calling frogs. While the morphology of the feeding apparatus is well studied in hematophagous Diptera that impact humans, frog-biting midges have received far less attention. We provide a detailed micromorphological examination of the piercing blood-sucking proboscis and maxillary palpus in three *Corethrella* species using scanning electron microscopy and histological semi-thin sectioning. We also compare the sensilla found on the proboscis tip and the palpus of *Corethrella* with other piercing blood-sucking Diptera. *Corethrella* spp. have a proboscis length of about 135 µm, equipped with delicate mandibular piercing structures composing the food canal together with the labrum and hypopharynx. Their proboscis composition is plesiomorphic and more similar to other short-proboscid hematophagous Culicomorpha (e.g., Simuliidae), in contrast to the phylogenetically more closely related long-proboscid Culicidae. As in other short-proboscid taxa, the salivary canal in *Corethrella* spp. transitions into an open salivary groove with one mandible forming a seal, whereas in Culicidae the salivary canal is closed until the tip of the proboscis. We discuss the possible functional constraints of very short, piercing blood-sucking proboscises (e.g., dimensions of host blood cells) that may limit the size of the food canal.

## 1. Introduction

Part of the success and diversity of insects stems from the large variety of niches they occupy; this is especially true for the type of food source they are specialized on [1]. Among the huge morphological and functional variety of fluid-feeding mouthparts, a piercing-sucking proboscis convergently evolved in many different insect taxa and represents a technique for feeding on food sources that are covered by a protective layer such as skin [2,3].

**Figure 1:**
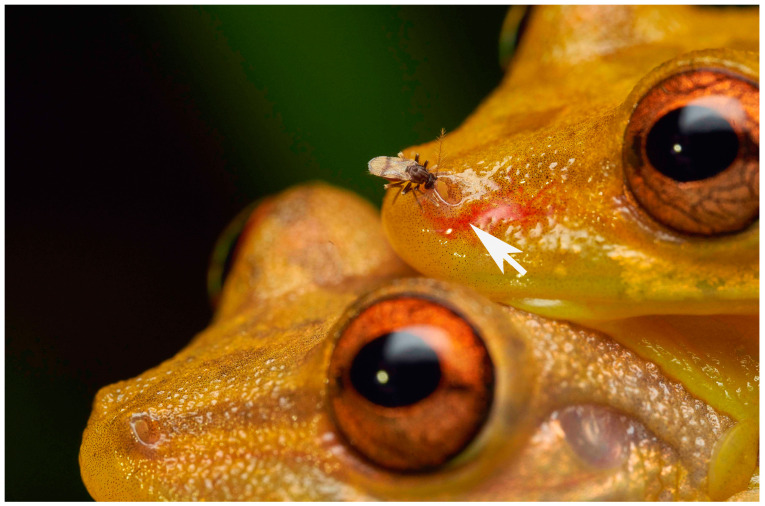
Female frog-biting midge (*Corethrella* spp.) sucking blood at the nostril of a male treefrog *Scinax elaeochrous* in amplexus; haematoma formed beneath the surface of the skin (**arrow**). La Gamba, Costa Rica. Photo: A. Ruppert.

Frog-biting midges (Corethrellidae, with the monotypic genus *Corethrella*) belong to the dipteran infraorder Culicomorpha, forming the superfamily Culicoidea together with the phantom midges (Chaoboridae), the meniscus midges (Dixidae) and the mosquitoes (Culicidae) [4,5]. To date, Corethrellidae comprises more than 120 described species [6,7,8,9]. The vast majority of *Corethrella* spp. likely remain unknown due to limited sampling effort, high beta diversity and the high probability of cryptic species [10,11]. *Corethrella* spp. are tiny (approx. 1–2 mm in body length), mostly tropical, blood-sucking flies whose females are specialized to feed on frogs which they locate and approach acoustically: the mating calls of male frogs serve as the main stimulus for far-range attraction [12,13]. Similar positive phonotactic behavior has been observed in other families of Diptera, including Culicidae [14], Psychodidae [15], Tachinidae [16] and Sarcophagidae [17]. It is likely that Corethrellidae also utilize additional cues to find their hosts such as chemical, visual and tactile indicators, as seen in other blood-sucking flies [11,18]. This is supported in *Corethrella* spp. by observed higher (non-acoustic) levels of host specialization, including the choice of host species and/or a specific feeding site on a host [19] (Figure 1). Like many other biting Diptera, female frog-biting midges rely on vertebrate (i.e., frog) blood to produce their eggs [12,18,20,21].

Although the form and function of fluid-feeding mouthparts in insects may vary depending on the utilized food source, the set of mouthparts always consists of the same principal components [2,22]. In blood-sucking Diptera, the feeding apparatus consists of the piercing–sucking proboscis and two sucking pumps: the cibarial pump beneath the clypeus and the pharyngeal pump in the posterior of the head [2,23,24,25]. In female Culicomorpha the proboscis is formed by the labrum, a pair of mandibles, a pair of maxillae, a hypopharynx and a labium; these typical components make up the ground plan features in adult lower Diptera [26]. These are modified from biting–chewing mouthparts in insects and enable hematophagous flies to pierce and to suck blood and/or other body fluids [22,27]. The labrum, the mandibles and maxillary laciniae are long, acute structures that are modified into thin piercing stylets that can penetrate the host together with the elongated hypopharynx [28]. The labium forms a trough-like sheath that partly surrounds the piercing stylets posteriorly [28], thereby forming a supporting and guiding structure for the piercing stylets [3,18]. The food canal, through which blood is sucked up, is formed by the labrum and hypopharynx; in some taxa the mandibles form part of the composition of the food canal. The salivary canal is formed by the hypopharynx and is used to inject saliva to counter the host’s mechanisms of blood clotting, platelet aggregation and vasoconstriction [3,23,29,30]. Since males do not feed on blood, mandibular piercing structures are reduced [31].

The aim of this study is to examine the proboscis morphology and micro-anatomy of the mouthparts of three species of *Corethrella* (Corethrellidae) and to compare these with other hematophageous Diptera to understand mouthpart evolution in the Culicomorpha. In this regard, we discuss the functional aspects of very short piercing mouthparts and the size limits of the feeding apparatus of blood-feeding insects.

## 2. Materials and Methods

### 2.1. Studied Species

*Corethrella* midges were caught using acoustic traps in Costa Rica at the Tropical Station of La Gamba (Golfito/Puntarenas, 83°12′7″ W, 8°42′2″ N; 77 m asl) and fixed in ethanol. Three *Corethrella* morphospecies, *C. ranapungens* Borkent, 2008, *C. amazonica* Lane, 1939, and *Corethrella peruviana* Lane, 1939, were studied. Light microscopic preparations of *Corethrella* midges (n = 10 of each species) were used for measuring body length, head height and proboscis length. The length of the proboscis was defined as the distance from the distal end of the clypeus to the apex of the labrum and measured using a light microscope (Nikon Labophot 2, Nikon, Tokyo, Japan) with an attached drawing device.

### 2.2. Semi-Thin Sections

For the semi-thin sections, *Corethrella* spp. were first dehydrated in ascending ethanol concentrations (70%, 80%, 90%, 95%, 100%; 15–30 min per step) and 100% acetone at room temperature. Specimens were then embedded in agar low viscosity resin (Agar Scientific Ltd., Essex, UK) after gradually increasing the concentration of the resin in three steps. After 2.5 h in a vacuum chamber at 150 mbar at 40 °C and hardening for 24 h at 70 °C, the resulting blocks were trimmed to an appropriate size and cut into 1 μm sections with a microtome (Leica EM UC6, Leica GmbH, Wetzler, Germany). The sections were mounted onto glass slides, stained with Toluidine blue (0.1%), embedded in agar low viscosity resin, and covered with cover glasses. Photos of the sections were taken using a microscope (Nikon Eclipse E800, Nikon, Tokyo, Japan) equipped with a digital camera (Nikon DS-Ri2, Nikon, Tokyo, Japan) at 400x magnification in an oil immersion. The contrast of the photomicrographs was adjusted using Adobe Photoshop (CC 2018, San Jose, CA, USA).

### 2.3. Scanning Electron Microscopy (SEM)

Preparation of the midges for the scanning electron microscope (SEM) started with dehydration in an ascending ethanol series (70%, 96%, 100% at room temperature; 30 min per step). To increase the durability of the animals against the electron beam, they were immersed in hexamethyldisilazane (HMDS) for 15 min at room temperature and then air- dried. The midges were then mounted onto the mounting tables using carbon platelets and conductive silver was used. Finally, specimens were coated with a thin gold layer using a sputter-coater (JEOL JFC-2300HR, JEOL Ltd., Tokyo, Japan). Photos of the specimens were taken using the scanning electron microscope Philips XL 30 ESEM (Koninklijke Philips N.V., Amsterdam, The Netherlands) at 15 kV voltage and JEOL JSM-IT300 (JEOL Ltd., Tokyo, Japan) at 20 kV voltage. Photos were edited and labelled using the Fiji plug-in ScientiFig. Measurements of the sensilla lengths were taken using the SEM photos of five specimens.

## 3. Results

### 3.1. Proboscis Morphology of Corethrella

**Figure 2:**
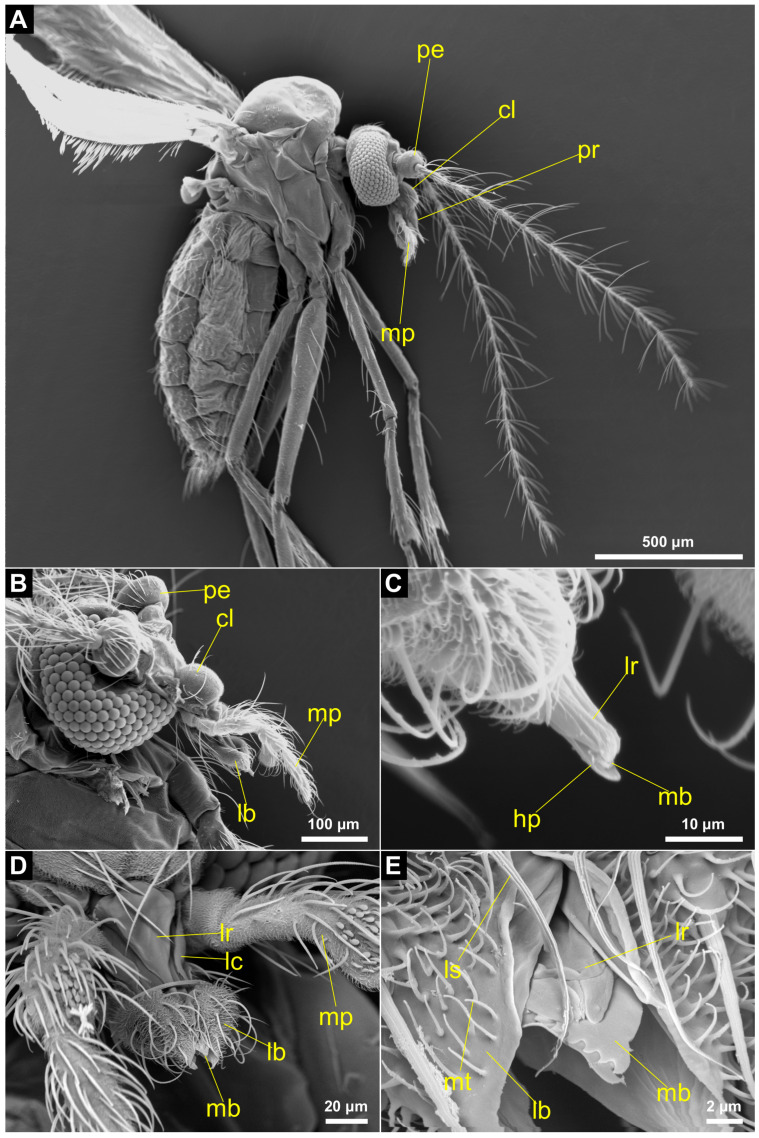
Feeding apparatus of female *Corethrella* (Corethrellidae, Diptera) (SEM). (**A**) Lateral view of *C. peruviana*. (**B**) Head and mouthparts of *C. ranapungens* in lateral view. (**C**) Proboscis tip of *C. ranapungens*; the mandibles are positioned between the labrum and hypopharynx (frontal view). (**D**) Maxillary palps and proboscis of *C. amazonica* in frontal view; the labella forms a sheath over the stylets. (**E**) Proboscis tip of *C. amazonica* (frontal view) highlighting the serrations on the mandibles and split labrum tip as well as microtrichia and grooved setae on the labella. cl: clypeus; hp: hypopharynx; lb: labellum; lc: lacinia; lr: labrum; ls: labellar seta; mb: mandible; mp: maxillary palp; mt: microtrichium; pe: pedicle; pr: proboscis.

The morphology of the proboscis is similar across the females of *C. peruviana, C. amazonica* and *C. ranapungens*. The proboscis is distinctly shorter than the head and measures about 1/10 of the body length (Figure 2A,B). The mean length of the proboscises ranges from 119.8 µm in *C. ranapungens* to 143.5 µm in *C. amazonica* (Table 1).

**Figure 3:**
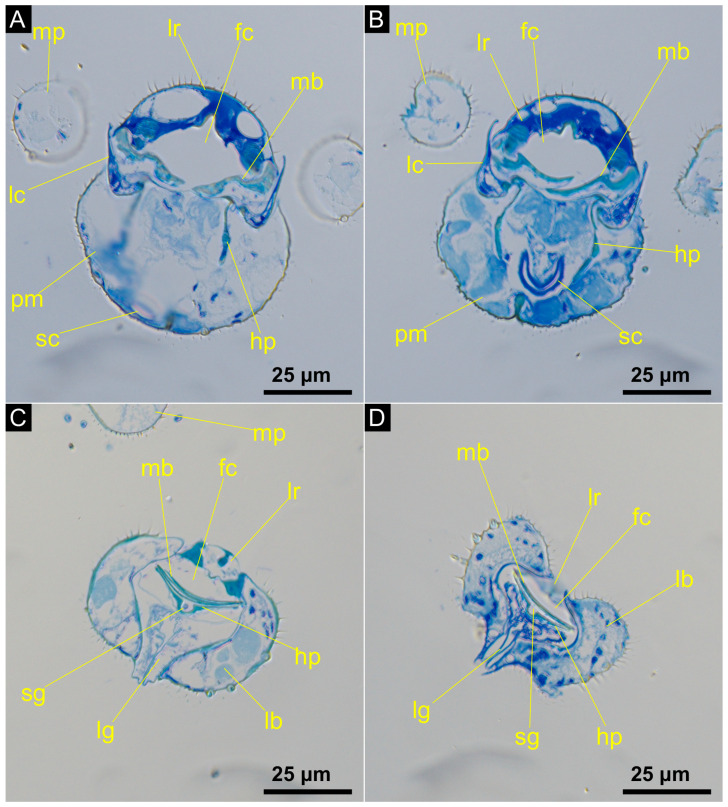
Cross-sections of the proboscis of *C. peruviana* (histological semi-thin sections). (**A**) In the proximal region the thin-walled salivary duct transitions into a thicker-walled salivary canal and (**B**) further distally into the salivary groove at the distal region of the proboscis (**C**,**D**). fc: food canal; hp: hypopharynx; lb: labellum; lc: lacinia; lg: ligula; lr: labrum; mb: mandible; mp: maxillary palp; pm: prementum; sc: salivary canal; sg: salivary groove.

**Figure 4:**
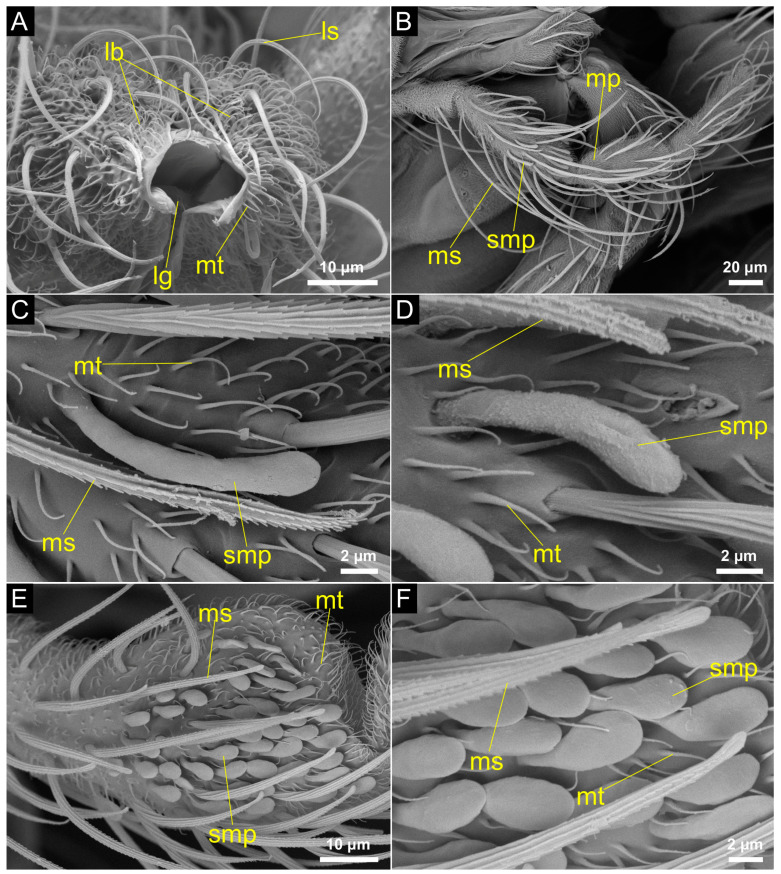
Microtrichia, setae and sensilla of the proboscis tip and maxillary palpus (SEM). (**A**) Proboscis tip of *C. amazonica* with microtrichia and socketed setae covering the labella. Ligula visible between the labella inside the opening of the food canal (ventral view). (**B**) Palpus of *C. peruviana* (lateral view). (**C**) Microtrichia, grooved, socketed and serrated setae and club-shaped sensilla on the maxillary palp of *C. peruviana* (lateral view). (**D**) Microtrichia, setae of various types and club-shaped sensilla on the palpus of *C. ranapungens* (lateral view). (**E**,**F**) Microtrichia, grooved, serrated setae and club-shaped sensilla on the palpus of *C. amazonica* (lateral view). lb: labellum; lg: ligula; ls: labellar seta; mp: maxillary palpus; ms: maxillary palpus seta; mt: microtrichium; smp: maxillary palpus sensillum.

The proboscis forms a piercing organ that includes the same morphological features found in most other Culicomorpha. The arched clypeus has numerous short microtrichia and a few long setae and its distal border is connected with the labrum. The elongated labrum, which does not have any setae, is slender towards the tip of the proboscis and forms the anterior border of the food canal (Figure 2C,D). At the apex of the proboscis the labrum splits into two parts, called labral pegs. The mandibles extend to the apex of the proboscis and lie very close to each other between the anterior labrum and the posterior hypopharynx (Figure 2C). One mandible covers the salivary groove, which is shaped by the hypopharynx. The mandibles and hypopharynx jointly form the food canal (approximately 25 µm in width) alongside the labrum (Figure 3). At the apex, the mouthparts form a small opening into the food canal that is 10 to 12 µm in width in *C. amazonica* (Figure 4A).

The mandibular stylets converge and overlap medially and lie very close to one another (Figure 2E and Figure 3C,D). One mandible forms the posterior cover of the food canal while the other covers the salivary groove (Figure 3C,D). The lateral edge of the tips of the mandibles are serrated. The teeth on the apex of the mandibles are evident in Figure 2E. The mandibular stylets, labrum and hypopharynx all reach to the very tip of the proboscis, whereas the laciniae, as part of the maxillae, extend only to two-thirds of the proboscis length and form the lateral borders of the food canal up to the middle region (Figure 3A,B). The laciniae appear as slender structures that converge distally, making them less pointed than the mandibles.

The labium is composed of the prementum in the proximal region of the proboscis and splits up into a paired labella and an unpaired ligula in the distal region. Here, the labella form a sheath surrounding the piercing organs (Figure 2 and Figure 3). Numerous long setae are found on the labella; these have grooves and smooth ridges along their longitudinal axes. The setae have pointed tips, extend from a socket and are 25 μm to 36 μm long (Figure 4A). There are also shorter setae, with a very similar appearance, but which are 11 μm to 14 μm in length. Short, curved structures (2 μm to 5 μm in length) without grooves or sockets are present in high abundance on the labella (Figure 4A). In the proximal region of the proboscis the hypopharynx lies anteriorly of the prementum and reaches into the prementum enveloping the salivary canal (Figure 3A,B). More distally, the salivary canal transitions into the salivary groove, which is formed by the hypopharynx (Figure 3C,D). Here, the hypopharynx becomes blade-shaped and serrated; it has the shape of a thin stylet. In the proximal region of the proboscis, the salivary canal is thick-walled (Figure 3B).

### 3.2. Maxillary Palpus

The palpi are longer than the proboscis but do not form part of the piercing apparatus itself (Figure 2B). They consist of five segments; the first two appear to be mostly fused (Figure 4B). The surface of each segment bears long socketed setae with grooves and serrated ridges along their longitudinal axes. The setae occur in two different length types: the longer setae measure between 93 μm and 143 μm in length, and the shorter vary in length between 22 μm to 48 μm (Figure 4B). The surface of the palpus is also covered with a large number of short and smooth microtrichia, which measure between 2 μm to 5 μm in length. The third segment is elongated and has blunt sensilla towards its distal end. These sensilla vary in their form and size across the three species of *Corethrella*. While they are longer and club-shaped in *C. peruviana* (Figure 4C) and *C. ranapungens* (Figure 4D), they have a spoon shape in *C. amazonica* and are shorter, more numerous and more dense (Figure 4E,F).

## 4. Discussion

### 4.1. Comparison of the Proboscis of Corethrella spp. to Other Hematophagous Diptera

Insects show a high degree of adaptation to the different kinds of food sources they use to nourish themselves [2]. In hematophagous insects a pattern of convergent evolution is seen where functional adaptation has led to a piercing and sucking proboscis that can pierce through the skin of vertebrates and suck up blood. Even though the piercing proboscises of different insect taxa may show different functional mechanisms, they still share functionally similar structures for puncturing, penetrating and anchoring the proboscis, a sheath-like covering component of the stylets and a food canal [3]. The main goal of this work was to analyze the structures that make up the piercing blood-sucking proboscis of the Corethrellidae using modern imaging techniques and to compare our findings with the existing literature [29,31]. From our detailed analysis, we found that all three species of *Corethrella* investigated displayed a very similar proboscis morphology to each other and which also matched with the known morphology of other species of Corethrellidae.

The short piercing structures of the proboscis in small-sized hematophagous Diptera, like the Corethrellidae, need to be rigid and stiff, as they are essential in the process of piercing vertebrate skin. In all blood-feeding Culicomorpha (e.g., frog-biting midges, mosquitoes, black flies, etc.), the labium forms a supporting structure around the piercing structures. Several species of Phlebotominae [3,32], Simuliidae [3,33,34] and Ceratopogonidae [3,35] show a similar proboscis morphology when compared to Corethrellidae, even though they are not closely related [4,5]. It is concluded that a proboscis shorter than the head is ancestral for Culicomorpha while the comparatively long proboscis of the relatively large Culicidae is derived. Likewise, we hypothesize that a scissor-like piercing mechanism including sideward movements of the mandibles [28,33,34] in non-culicid Culicomorpha is plesiomorphic and the pro- and retraction of the mandibles observed in Culicidae [23,36,37] is apomorphic. A putative functional adaptation of the proboscis of Corethrellidae to frogs are the serrated mandibles and unarmed laciniae. Borkent (2008) [11] points out that this combination of traits can also occur in species of Psychodidae and Ceratopogonidae that obtain their blood meals from anuran hosts, and that these species may not necessarily need to rely on the anchoring function of armed laciniae, possibly because of the loose skin of their hosts. In *Corethrella* spp., the food canal is bordered anteriorly by the labrum and posteriorly by the mandibles, with the hypopharynx lying further along the posterior side. It is likely, however, that the hypopharynx also plays an important role in forming the food canal together with the mandibles. This is necessary because the movement of the mandibles during the piercing process of the host’s skin interferes with the sealing of the food canal. In Simuliidae [33,34] and Ceratopogonidae [38], the mandibles perform a snipping motion to open the skin of the host. Even though the exact mechanism of the skin-piercing process in Corethrellidae is unknown, the absence of an interlocking structure seems to indicate that the mandibles do not move in such a way [31,39]. However, they probably do move to some degree during the piercing process since serration is evident in the studied species. Figure 1 indicates that blood-feeding *Corethrella* midges can cause a haematoma beneath the skin of the frog and that they will suck blood from the pool of escaped blood, as do many other short-proboscid hematophagous lower Diptera [18,33].

Some interesting differences of the proboscis morphology are found in *Corethrella* when compared to representatives of the Culicidae. Firstly, the proboscis of mosquitoes is much longer when compared to body size than in *Corethrella* and can penetrate the host’s skin deeper [18]. Secondly, the food canal is mainly formed by the labrum and is bordered ventrally by the hypopharynx, but the mandibles and laciniae lie outside of the food canal and not in between the labrum and the hypopharynx [3,23,40,41]. The overlapping mandibles, plus the hypopharynx together with the labrum, form the food canal in Corethrellidae [29,31], similar to Ceratopogonidae [35], Phlebotominae [3,32] and Simuliidae [3,33,34]. Thirdly, the salivary canal of Culicidae is closed to the tip of the long proboscis where it releases salivary fluid during the piercing process [3,23,41,42,43]. In contrast, the salivary canal in Ceratopogonidae, Phlebotominae and Simuliidae opens and transitions into the salivary groove towards the tip of the proboscis; a similar morphology is also found in *Corethrella*. Furthermore, in the proximal region of the proboscis of these taxa one mandible forms a closure of the salivary groove [29]. These shared features in Corethrellidae, Phlebotominae, Ceratopogonidae and Simuliidae are the plesiomorphic condition whereas the character states found in Culicidae are autapomorphic.

### 4.2. Sensilla of Labella and Maxillary Palpus

Three different types of sensilla were observed on the labella and the palpi of the females of *Corethrella*. Their long, grooved appearance, as well as their placement in a socket, suggests a function as a mechanoreceptor [44]. No pores or openings, which would suggest a chemosensory function, could be found. Two types of long setae that differ in length were found on the labella of the *Corethrella* species investigated. Both types resemble the long setae found on the palpus of *Corethrella* and show similar grooved surfaces and a socket. Lee and Craig (2009) [45] found two length types of long setae on the labella of *Aedes aegypti* (Culicidae). They state that the longer type, called long labellar hairs, are mechanosensory and are probably responsible for checking the position of the labella on the host’s skin during the feeding process. The shorter setae, called medium-sized hairs, are chemosensory and might serve to probe the skin of the host to check for suitability. Spiegel et al. (2005) [46] found three different length types of this sensillum on the labellar lobes and maxillary palps of *Lutzomyia longipalpis* (Psychodidae) and described them as trichoid sensilla. Even though the authors were not able to detect pores or openings on the surface of these sensilla using SEM, they found indirect proof of their existence by using a silver staining method. This would suggest a chemosensory function of these sensilla. In the investigated species of *Corethrella*, the short, very abundant cuticular structures on the labella and the palpus closely resemble the microtrichia of species of Psychodidae [46] and Culicidae [45]. Electrophysiological and ultrastructural studies are needed for a profound comparison of the function of the mouthpart sensilla in the various taxa of Culicomorpha.

The club shaped sensilla on the third segment of the palpus observed in the investigated species of *Corethrella* are similar to those found in species of Simuliidae [47] and Ceratopogonidae [35,38], as well as other species of Corethrellidae [29]. Spiegel et al. (2005) [46] described similar sensilla on the palpus of *L. longipalpis* (Psychodidae) as capitate peg sensilla. In Culicidae, these sensilla have a known sensitivity to carbon dioxide [48,49]. These sensilla most likely also assist frog-biting midges in locating their hosts by detecting the carbon dioxide released by the frog in addition to other recognition cues, such as skin peptides [50]. The possible use of additional olfactory or gustatory and visual cues appears congruent with observations on host-seeking behavior, host preferences and feeding site selection in *Corethrella* [11,19,51].

### 4.3. Impact of Proboscis Size

Anuran erythrocyte blood cells are relatively large and range from 10.6 µm to 28.3 µm in diameter among different species [52]. There are only a few published photos of histological cross sections of a hematophagous insect’s proboscis [3] which can be used to estimate whether the size of the blood cells of a host can possibly limit the dimensions of blood feeding organs. In Corethrellidae, the size of blood cells of anuran hosts must be wide enough to enable the blood cells to pass through the food canal during the feeding process. In the present study, SEM photos of *C. amazonica* indicate an apical proboscis opening of about the lower range size of anuran blood cells. In the studied *Corethrella* species the cross sections of the food canal fit the larger range size of anuran blood cells. Similarly, the erythrocyte blood cell size of mammals [53] and birds [54] may have a size constraining effect on the mouthparts of hematophagous Simuliidae, Ceratopogonidae and Culicidae. However, it is not known whether the smaller diameter of the blood cells of mammals, i.e., 2.1 µm to 10.8 µm [53] would allow for narrower feeding organs.

De Silva et al. (2014) [39] discussed the relationship between the vascularization density and depth of blood vessels in the skin of anuran hosts to the preferred feeding site of female Corethrellidae. By measuring the length of the labium, the authors estimated the potential depth frog-biting midges would need to penetrate to reach the blood vessels. However, the labium does not penetrate the skin during blood-feeding. In the present study, the length of the proboscis of the investigated species of *Corethrella* was determined by measuring the distance from the distal end of the clypeus to the tip of the labrum. Since the clypeus is not inserted into the hosts skin during the feeding process this marks the maximal insertion depth of the proboscis of about 120–145 µm depending on the species, and thus the maximal depth in which blood vessels can be reached. As the preferred anuran hosts of the studied *Corethrella* species are known [11], the experimental framework of the study of de Silva et al. (2014) [39] could potentially be applied to the results of this work. However, skin measurements for the target frog host species are missing; future research would need to acquire this data to test de Silva et al.’s (2014) [39] hypothesis. Data on skin thickness and varying vascular properties may also help explain the presence of preferred feeding sites on anuran hosts. However, other factors may offset skin thickness such as when anticoagulant substances cause a blood pool to form under the skin which is then reachable even when using a very short proboscis. Such superficial blood pools have been observed during and after the blood meal of frog-biting midges (J.V. personal observation). Future comparative studies should investigate the interplay between proboscis length, depth of penetration, skin thickness at different feeding sites and the role of anticoagulants in various hematophagous insects.

## Figures and Tables

**Table 1:** Body length, head height (dorso-ventral length of complex eyes) and proboscis length (clypeus to tip of the labrum) in female *Corethrella* species; means ± SD, n = 10 individuals per species; measurements in µm, rounded to one decimal place.

*C. ranapungens*			*C. amazonica*			*C. peruviana*		
Body	Head	Proboscis	Body	Head	Proboscis	Body	Head	Proboscis
1326.9	258.1	119.8	1224.7	307.2	143.5	1513.4	320.8	141.2
±159.3	±9.2	±10.1	±45.3	±14.8	±10.6	±91.7	±12.8	±10

## Data Availability

The histological sections and SEM specimens are available on request from the corresponding author.
